# Dissociable effects of mild COVID-19 on short- and long-term memories

**DOI:** 10.1093/braincomms/fcae270

**Published:** 2024-08-14

**Authors:** Lauren Z Atkinson, Jude L Thom, Anna Christina Nobre, Nahid Zokaei

**Affiliations:** Department of Psychiatry, Oxford Centre for Human Brain Activity, Wellcome Centre for Integrative Neuroimaging, University of Oxford, Oxford OX3 7JX, UK; Department of Experimental Psychology, University of Oxford, Oxford OX2 6GG, UK; Department of Psychiatry, Oxford Centre for Human Brain Activity, Wellcome Centre for Integrative Neuroimaging, University of Oxford, Oxford OX3 7JX, UK; Department of Experimental Psychology, University of Oxford, Oxford OX2 6GG, UK; Department of Psychiatry, Oxford Centre for Human Brain Activity, Wellcome Centre for Integrative Neuroimaging, University of Oxford, Oxford OX3 7JX, UK; Department of Experimental Psychology, University of Oxford, Oxford OX2 6GG, UK

**Keywords:** short-term memory, long-term memory, COVID-19, cognitive deficit, memory precision

## Abstract

Recent studies have highlighted the presence of cognitive deficits following COVID-19 that persist beyond acute infection, regardless of the initial disease severity. Impairments in short- and long-term memory are among the core deficits reported by patients and observed in objective tests of memory performance. We aimed to extend previous studies by examining performance in a task that allows us to directly compare and contrast memories at different timescales. More specifically, we assessed both short- and long-term memories for contextual–spatial associations encoded during a common session and probed at different durations using an equivalent task in non-hospitalized individuals recovering from mild COVID-19 compared to healthy controls. The approach equated all aspects of memory materials and response demands, isolating performance changes resulting only from memory timescales and thus allowing us to quantify the impact of COVID-19 on cognition. In addition to providing measures of accuracy and response times, the task also provided a sensitive continuous readout of the precision of memory representations, specifically by examining the resolution with which spatial locations were retained in memory. The results demonstrated selective impairment of long-term memory performance in individuals recovering from mild COVID-19 infection. Short-term memory performance remained comparable to healthy controls. Specifically, poor precision of long-term memory representations was demonstrated, which improved with days since diagnosis. No such relationship was observed for short-term memory performance. Our findings reveal a specific impairment to the precision of spatial–contextual long-term memory representations in individuals recovering from mild COVID-19 and demonstrate evidence of recovery in long-term memory over time. Further, the experimental design provides a carefully controlled and sensitive framework to assess memory across different durations with the potential to provide more detailed phenotyping of memory deficits associated with COVID-19 in general.

## Teaser

Atkinson *et al*. report a selective impairment in long-term but not short-term memory performance in individuals recovering from mild COVID-19 infection, which improved with days since diagnosis. Memory was examined in a well-controlled task for contextual–spatial associations utilizing equivalent stimulus materials and response demands for different memory durations.

## Graphical Abstract

**Graphical Abstract:**
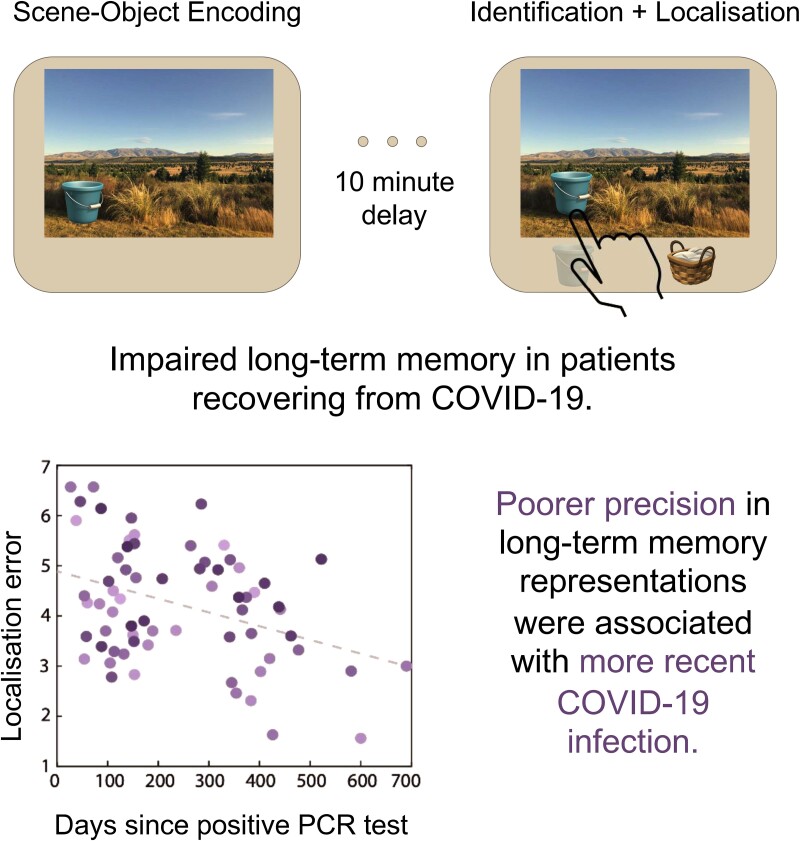

## Introduction

Although COVID-19 is primarily a respiratory disease, there is significant concern surrounding the burden associated with cognitive impairment that persists beyond acute infection.^1^ These cognitive deficits include impaired attention, memory and executive function, which can persist for weeks or months, regardless of acute COVID-19 severity.^2-6^ Importantly, both individuals with acute COVID-19 and those who experience ongoing and fluctuating physical and mental symptoms, often referred to as post-COVID-19 syndrome or Long-COVID, have reported experiencing impaired cognition.^6-13^ The proportion of individuals with persistent symptoms can vary from 10 to 33%.^10,14-16^

Impairments in short- (STM) and long-term memory (LTM) are among the core cognitive deficits regularly reported in individuals following COVID-19 infection.^2,17,18^ Objective memory deficits (as part of a composite cognitive score) have been reported in individuals who recovered from COVID-19 (without Long-COVID), present across all magnitudes of severity at the time of infection;^19^ but particularly in those who were hospitalized.^20^ Similarly, in a large sample of patients with Long-COVID, self-reported memory concerns were shared across all age groups in ∼73% of individuals.^11^

Specific LTM impairments have been reported in individuals who recovered from COVID-19 with mild symptoms at the time of infection.^21^ In a recent online study, recovered individuals had a significantly larger memory decrement in an object-recognition task after a 30 min delay, which was observed even 6 months following acute infection.^21^ However, the presence of STM impairments post–COVID-19 infection is unclear. Most studies fail to observe deficits in STM compared to LTM when using a neuropsychological assessment^19-21^ despite significant self-report concerns following COVID-19 infection.^22^

Notably, objective memory deficits, specifically in hospitalized patients and those recovering from mild COVID-19, have only been unmasked by targeted memory tests rather than relying on commonly used neuropsychological or self-report measures that lack the sensitivity to detect subtle deficits.^23^ The development of sensitive memory tests to better map the cognitive deficits associated with COVID-19, even in younger adults, is currently an unmet clinical need. This is particularly important considering that a large proportion of the population will recover from acute or sub-acute symptoms of the disease but may experience prolonged, undetected cognitive deficits or be prone to neurodegeneration, possibly due to the development of cerebrovascular disease as a result of COVID-19 infection.^24-27^

Here, we used recently developed tasks^28^ assessing short- and long-term memories to identify, characterize, and separate specific impairments in short- and long-term memory associated with COVID-19. The tasks possess a high level of experimental control, utilizing equivalent stimulus materials and response demands by using a common encoding phase to assess memory at both short and long durations for the same type of contextual–spatial associations. Additionally, both tasks provide comparable measures of the precision of memory representations, specifically by examining the resolution with which locations were retained, using continuous analogue measures of location memory. These measures have proven successful in quantifying the reliability and quality of memory in healthy ageing, neurodegenerative disorders and at-risk populations^28-32^ and contrast with standard measures, which use discrete binary (correct/incorrect) responses.^23,33^ Additionally, the present tasks have the potential to isolate impairments that may be attributed to the maintenance of particular types of information across timescales (i.e. remembering a location irrespective of duration), types of processes (short- versus long-term memory) or both, which in turn will inform the possible underlying mechanisms associated with memory deficits following mild COVID-19 infection.

### Participants

The study was approved by the Medical Sciences Interdivisional Research Ethics Committee of the University of Oxford and complied with the Declaration of Helsinki. In total, 140 participants took part in this study and performed the tasks in the period between September and December 2021. Participants were recruited via the Prolific online recruitment platform (www.prolific.co) and performed the task remotely. They gave written consent and were reimbursed at £7.5 per hour. All participants had normal or corrected-to-normal vision and normal colour vision.

Seven of the 140 participants were excluded from analysis. Of these, three reported a history of mental/neurological illness and four were experiencing long-term effects of COVID-19 (self-diagnosed Long-COVID based on self-report measures). Even though Long-COVID is not equivalent to mental/neurological illnesses, patients with persistent Long-COVID were excluded as cognitive symptoms related to this may present differently than those observed in individuals recovering from mild COVID-19. Furthermore, participants in the COVID-19 cohort could only enrol in the study if they reported having contracted COVID-19 which was confirmed by a polymerase chain reaction test but not being hospitalized due to COVID-19. Similarly, in the control group, only participants who reported not having contracted COVID-19 themselves, or being exposed to by a household member, were allowed to participate in the study.

The final cohort consisted of 67 participants who had previously contracted COVID-19 [age range, 20–60; days since diagnosis, 27–690 days; standard deviation (std), 158.5; 41 female] and 66 control participants who had never knowingly contracted COVID-19 (age range, 19–59; 37 female; see Table 1 for demographic information). Participants reported experiencing minimal physical symptoms related to COVID-19 (see Supplementary Fig. 1). There was no significant difference between years in full-time education between the two groups [*t*(131) = 1.142, *P* = 0.25].

**Table 1:** Demographics and questionnaire data for patients recovering from COVID-19 and healthy controls

	Recovering from COVID-19 (*n*=67)	Healthy controls (*n*=66)
Age: range	20–60	19–59
Gender: *n* female	41	37
Days since diagnosis: range	27–690	n/a
Education: mean (std)	15 (3.9)	14.7 (4.5)
Family history of dementia (siblings, parents or grandparents): *n*	12	18
Family history of stroke (siblings, parents or grandparents): *n*	6	10
Smoking: *n*	3	6
CFQ: mean (std)	19.9 (13.6)	20.9 (12.9)
AMI: mean (std)	2.1 (0.6)	2.07 (0.7)
GAD-7 General: mean (std)	3.6 (2.7)	3.3 (2.5)

Participants also completed questionnaires that included family history of dementia or stroke (n.s. difference between the two groups), the Apathy-Motivation Index (AMI), the Cognitive Failure Questionnaire (CFQ), and the Generalized Anxiety Disorder Assessment (GAD-7). There was no significant difference in these questionnaires between recovering patients and healthy controls (Table 1).

## Methods

**Figure 1:**
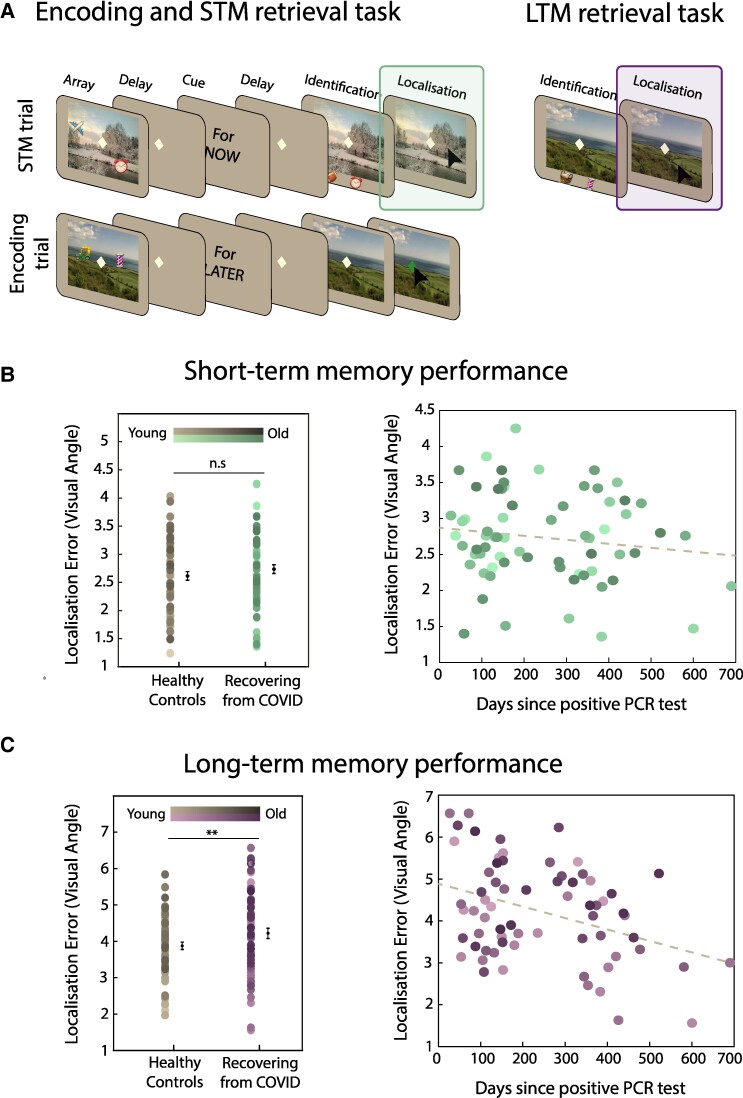
**(A) Short- and long-term memory tasks.** Each trial in the encoding and the STM (left panel) task started with a memory array of two objects embedded within a scene, followed by a delay before the presentation of the duration cue. After the presentation of a delay, in the STM trials, memory scene appeared and, after a delay, two objects appeared underneath. Participants selected the object they had seen in the memory array (identification) and indicated its original location (localization) as precisely as possible. In the encoding trials, the delay was followed by the presentation of the scene without the objects and participants had to click the fixation as soon as it turned green. The LTM retrieval task (right panel) was like the STM retrieval phase; a previously studied scene (from the encoding trials) appeared alongside two objects. Participants had to identify the object associated with the scene and localise it as accurately as possible. (**B**) Short-term memory performance. Individuals recovering from COVID-19 performed similarly to healthy controls [left panel, ANOVA, *F*(1130) = 1.74, *P* = 0.19], and their performance did not relate to days since their diagnosis (right panel, *r* = −0.141, *P* = 0.256). (**C**) Long-term memory performance. Individuals recovering from COVID-19 performed significantly worse than healthy controls [left panel, ANOVA, *F*(1130) = 5.06, *P* = 0.026]. There was a significant relationship between their LTM performance and days since diagnosis, with better performance in individuals with increasing days since diagnosis (*r* = −0.376, *P* = 0.002). Each dot represents an individual participant, and different shades of colour represent the ages of the participants.

The task was programmed with PsychoPy (https://www.psychopy.org/) and hosted on Pavlovia (https://pavlovia.org/). Participants were first asked to keep in mind, and later to report, both the identity and location of an object associated with a scene. Memory reports occurred after either a short (short-term memory task—Fig. 1A, left panel) or a long (long-term memory task—Fig. 1A, right panel) delay. The methods for this task are almost identical to those previously described by Čepukaitytė and colleagues,^28^ but we briefly summarize them again below in order to highlight small adaptations necessary in order to run the task as an online study.

### Stimuli

Stimuli consisted of photographs of indoor and outdoor scenes and images of everyday objects. The scenes (20 × 20° of visual angle at 30 cm distance) were selected from an in-house repository of coloured photographs and copyright-free images. The objects (1.35 visual degrees at an approximate distance of 60 cm) were selected from emoticons of everyday household and clothing items, food, toys and sports equipment (https://emojipedia.org/). For each participant, 44 scenes were randomly drawn from a pool of 399, and 132 coloured objects were randomly drawn from a pool of 135. Objects were positioned randomly within scenes, but with the following constraints: objects were at least one visual degree away from the centre and edges of the screen, a minimum distance of 2.5 visual degrees separated the centre of the objects and objects could appear in all four quadrants of the scene with equal probability.

### Tasks and procedure

At the start of the experimental session, participants’ screen resolution was estimated by asking them to adjust an image of a credit card to match the size of a physical credit card. We could thereby calculate the ratio between the card image width in pixels and the actual card width in centimetres. This allowed us to estimate the pixel density (i.e. pixel per centimetre). By instructing participants to view the monitor at one arm’s length (∼60 cm), it was possible to control the size of the stimuli in degrees of visual angle in approximate terms.^34^

### Short-term memory/encoding task

In the first stage of the task (Fig. 1A, left panel), participants were presented with a memory array that consisted of two objects embedded within a scene (5 s). Participants were asked to find and remember both the identity and location of both objects. The memory array was followed by a blank delay for 1 s. This was followed by a duration cue—‘For Now’ or ‘For Later’—presented at the centre of the screen (0.5 s). The duration cue indicated the duration participants had to keep the information in mind.

In trials containing the ‘For Now’ cue, participants were probed after a 3.5 s blank delay (Fig. 1A, left panel, STM trial). At probe, the scene from the memory array reappeared with two objects below. Only one of the objects had previously been embedded in the scene (target), while the other had not (foil). Participants were instructed to identify the target object as quickly as possible (identification; pressing the keyboard letter C or M for left and right, respectively) and click the object’s original location within the scene (localization), prioritizing precision over response time when reproducing its location. Once satisfied with their response, participants pressed the mouse button to proceed to the subsequent trial.

In trials containing the ‘For Later’ cue, the cue was followed by a delay (3.5 s) and the presentation of the scene (Fig. 1A, left panel, Encoding trial). In these trials, participants had to click the fixation cross as soon as it turned green (after 1 s) to proceed to the subsequent trial.

This task consisted of two blocks of 20 trials each. Trial types (‘For Now’ and ‘For Later’) were randomly intermixed across blocks. All participants completed four practice trials to familiarize themselves with the procedures before the task began. Practice trials consisted of a separate set of scenes and objects.

### Long-term memory

Participants completed the LTM task (Fig. 1A, right panel) after a break of ∼10 min during which they filled out questionnaires concerning demographic variables, general health, family history of dementia and stroke, smoking habits, cognitive failures and psychological traits related to mood and motivation (Table 1). The LTM retrieval stage was identical to that used earlier for the STM retrieval. In each trial, a scene previously presented during the Encoding trials appeared with two objects displayed underneath. Using the same procedures as in the STM trials, participants were instructed to identify the target object (identification) and its exact location (localization) within the scene. The task consisted of one block of 20 trials.

### Statistical analysis

Data were pre-processed and analysed using Matlab release 2018. Trials were excluded from the analysis if reaction times to identify objects were faster than 100 ms or localization of objects occurred outside boundaries of the scene. In total, ∼1% of trials were excluded. Additionally, only trials in which targets were correctly identified were included in the analysis of identification times and localization errors.^28^

Repeated-measures ANOVAs with duration cue as a within-subject factor, COVID-19 status as a between-subjects factor and age as a covariate were conducted for identification accuracy, identification response times and localization error (statistical threshold = 0.05). Data that were not normally distributed were transformed, allowing the use of parametric statistics. Accuracy data were transformed using arcsine transformation and response times were log-transformed. Lastly, to explore the relationship between STM and LTM, multiple regression models were fitted using mean LTM localization error data.

## Results

Means and stds for different variables are presented in Table 2. Accuracy on the identification tasks showed that performance decreased with age (significant main effect of age: *F*(1130) = 4.93, *P* = 0.028, η^2^_p_ = 0.04), and overall, participants performed worse in the LTM compared to STM task (significant main effect of memory duration: *F*(1130) = 7.67, *P* = 0.006, η^2^_p_ = 0.06). There was no main effect of COVID-19 status or interactions among any of the factors. Similarly, identification response times increased with increasing age (significant main effect of age: *F*(1130) = 13.8, *P* < 0.001, η^2^_p_ = 0.1) and were higher in the LTM task compared to the STM task (significant main effect of memory duration: *F*(1130) = 7.27, *P* = 0.008, η^2^_p_ = 0.05). There was no main effect of COVID-19 status or interactions among any of the factors.

**Table 2:** Means and standard deviations for all experimental variables

	Recovering from COVID-19	Healthy controls
Short-term memory: mean (std)		
Identification accuracy	0.95 (0.06)	0.94 (0.07)
Identification times (s)	2.1 (0.66)	2.3 (0.87)
Localization error	2.7 (0.62)	2.6 (0.62)
Long-term memory: mean (std)		
Identification accuracy	0.84 (0.1)	0.85 (0.1)
Identification times	3.4 (1.32)	3.5 (1.2)
Localization error^a^	4.3 (1.15)	3.8 (0.84)

^a^Significant difference.

Localization error, the difference between the selected response location and the true location of the object within the scene, increased with increasing age (significant main effect of age: *F*(1130) = 9.07, *P* = 0.003, η^2^_p_ = 0.07) and was higher in the LTM task compared to the STM (significant main effect of memory duration: *F*(1130) = 5.46, *P* = 0.021, η^2^_p_ = 0.04). Additionally, there was a significant main effect of COVID-19 status group on performance (*F*(1130) = 5.98, *P* = 0.016, η^2^_p_ = 0.044). There were no interactions among any of the factors.

To explore the effects of COVID-19 on short- and long-term memories independently, we conducted subsidiary ANOVAs for each memory duration separately (with age as a covariate). There was no difference in STM performance between individuals recovering from COVID-19 and controls (*F*(1130) = 1.74, *P* = 0.19—Fig. 1B, left panel). Individuals recovering from COVID-19 however performed significantly worse than controls in the LTM task (*F*(1130) = 5.06, *P* = 0.026—Fig. 1C, left panel).

To explore the relationship between COVID-19 and memory performance further, we examined the relationship between localization performance at both memory durations and days since COVID-19 diagnosis was confirmed with a positive PCR test. There was a significant relationship between days since diagnosis and LTM performance (*r* = −0.376, *P* = 0.002—Fig. 1C, right panel), with localization error reducing systematically with increasing days since diagnosis. In contrast, days since COVID-19 diagnosis did not correlate with STM performance (*r* = −0.141, *P* = 0.256—Fig. 1B, right panel). Additional analysis on the effect of age on the relationship between days since diagnosis and LTM performance was not significant.

Lastly, to explore whether variations in STM localization error and age predicted long-term memory performance, we used a GLM. A model including age and STM performance was a significant predictor of LTM localization performance (*F*(2, 66) = 8.23, *P* = 0.001, *R2* = 0.2). Both variables contributed significantly to the prediction (*P* < 0.05). Participants with a larger STM error and of increasing age were more likely to produce a larger LTM localization error.

## Discussion

In the present study, we examined short- and long-term memory performance in individuals who recovered from COVID-19 compared to healthy controls. The COVID-19 group had recovered from mild infections and were not hospitalized, nor had they sought medical help at the time of diagnosis. Memory for both short and long durations was assessed with a high level of experimental control by using identical stimuli and response demands and, importantly, by employing a comparable encoding phase, which allows for a direct comparison of memory at both durations.

The results demonstrate impaired LTM performance in individuals recovering from mild acute COVID-19 infection, while their STM performance remained comparable to healthy controls. Specifically, poor precision of LTM was demonstrated when participants were asked to recall the exact location of an object within a scene. There were no differences in the accuracy or response times of LTM when participants had to identify which object had previously appeared in the scene. Notably, we found that the precision of LTM performance improved with days since diagnosis highlighting a relationship with COVID-19. No such relationship was observed for STM performance. Future studies should examine the effect of age of participants on this relationship with larger numbers of participants.

Our findings build on related previous studies reporting impaired LTM in patients with both mild and severe COVID-19 infections.^19-21,35,36^ In a recent study by Zhao and colleagues,^21^ young participants who had recovered from mild COVID-19 performed a battery of cognitive tasks that included memory, executive function and planning tests. Even though their accuracy in immediate memory was intact, there was a significant increase in memory decrement over 30 minutes in young participants who had recovered from COVID-19 compared to age-matched controls. Interestingly, recovered young participants performed at levels comparable to older healthy adults, and their memory decrement, like in our study, was related to days since diagnosis. In another study using an identical task for both memory durations, participants previously hospitalized with severe COVID-19 showed a similar pattern of memory performance with an increased decrement in LTM memory while their STM was preserved.^20^

Extending this rapidly growing literature with a carefully controlled study that allows for direct comparison between memories at different durations and provides a more sensitive measure of memory resolution, our results demonstrate that the precision with which information is retained over long durations is impaired in participants recovering from mild COVID-19, while their STM memory is preserved. Importantly, this impairment was not explained by changes in LTM performance with age. In a previous study using an identical task design,^28^ we have shown changes to LTM performance with advancing age. In addition to age-related changes to memory, here we reveal that COVID-19 impacted LTM performance, regardless of the age of participants. Expanding on these findings, future studies should focus on relating impaired LTM performance to performance in other cognitive functions and various clinical measures, to provide a better understanding on the nature and impact of this deficit.

Unlike previous studies, short- and long-term memories were tested using different sets of equivalent stimuli while matching encoding and response demands, minimizing interference across memory durations. Immediate recall of items from STM can improve long-term memories;^37-40^ this can make it challenging to interpret the exact nature of increased memory decrement following longer delays (e.g. 30 minutes) in previous studies. However, by separating short- and long-term memory trials, the current findings point towards a specific deficit observed only in LTM. Additionally, our study provided measures of memory accuracy, precision, and response times, with COVID-19 impacting only the resolution with which locations were retained in memory. This contrasts with other studies that have reported slowness of response times across the board in individuals recovering from COVID-19.^18-20,41^ Here, we show that impairments in LTM can occur independently of a global change in response times. Instead, it may reflect a selective impairment in LTM alone. Lastly, memory precision, here indexed by localization error, provides a sensitive measure of memory performance, which captures subtle changes in memory performance even at shorter delays.^23,30,42^

There are multiple mechanisms by which COVID-19 infection can result in the observed specific cognitive deficits in LTM performance. In a longitudinal study by Douaud and colleagues,^43^ one of the most consistent abnormalities observed was in the left parahippocampal gyrus, a brain region crucial for contextual episodic memories. The parahippocampal gyrus showed a greater reduction in grey matter thickness and increased tissue damage due to COVID-19 infection compared to any change due to normal ageing. In addition to these observed changes, other factors that are not necessarily mutually exclusive can result in neurological dysfunction that, in turn, can impact LTM performance. These can include viral encephalitis, neuroinflammation, cerebrovascular disease, cerebral microvascular injury or hypometabolism, all of which have been observed in patients with COVID-19;^44-47^ though the exact nature of the relationship between these brain changes and cognitive deficits in general remains to be elucidated.

Unlike some studies,^11,18,48,49^ the current results showed that STM remained intact following COVID-19 infection. Whilst prior research has found evidence for STM deficits, specifically in Long-COVID patients, these have used either self-report measures or neuropsychological assessments [e.g. sub-items of the Montreal Cognitive Assessment (MoCA)] that lack sensitivity to subtle changes in memory.^23^ To date, however, no study has explored whether persistent self-reported STM concerns in Long-COVID patients remain when using objective and sensitive memory measures. Such a study can aid in a more accurate delineation of the similarities and differences between acute and long COVID-19 and their possibly distinct effects on cognition, such as those observed in STM performance. In fact, studies that report impaired STM following acute COVID-19 infection do not exclude participants that report experiencing ongoing symptoms^11,18,48,49^ while any study that excludes Long-COVID patients fails to observe any impairments in STM.^19-21^

Our experimental design, with matching encoding and response demands for memories at short and long delays, provides a carefully controlled and sensitive task with the potential to explain the exact nature of deficits associated with both acute and Long-COVID. As the pandemic progresses, there is a need for more detailed phenotyping of deficits associated with COVID-19. Using sensitive and selective tasks to various cognition aspects will shape our understanding of related disorders, aid in the development of targeted medication and help track disease and treatment progression. This easy-to-administer online task provides such an opportunity, specifically to examine memory function at different timescales.

## Supplementary Material

fcae270_Supplementary_Data

## Data Availability

Task scripts and data are available upon request.
