# Supplementary material for: Dissociable effects of mild COVID-19 on short- and long-term memories
Source: Brain Commun. 2024 Aug 14;6(4):fcae270. doi: 10.1093/braincomms/fcae270 (PMC11358641; doi:10.1093/braincomms/fcae270)

Supplementary Figure 1. Frequency of responses on a VAS scale on different physical symptoms commonly linked to COVID-19 infection.

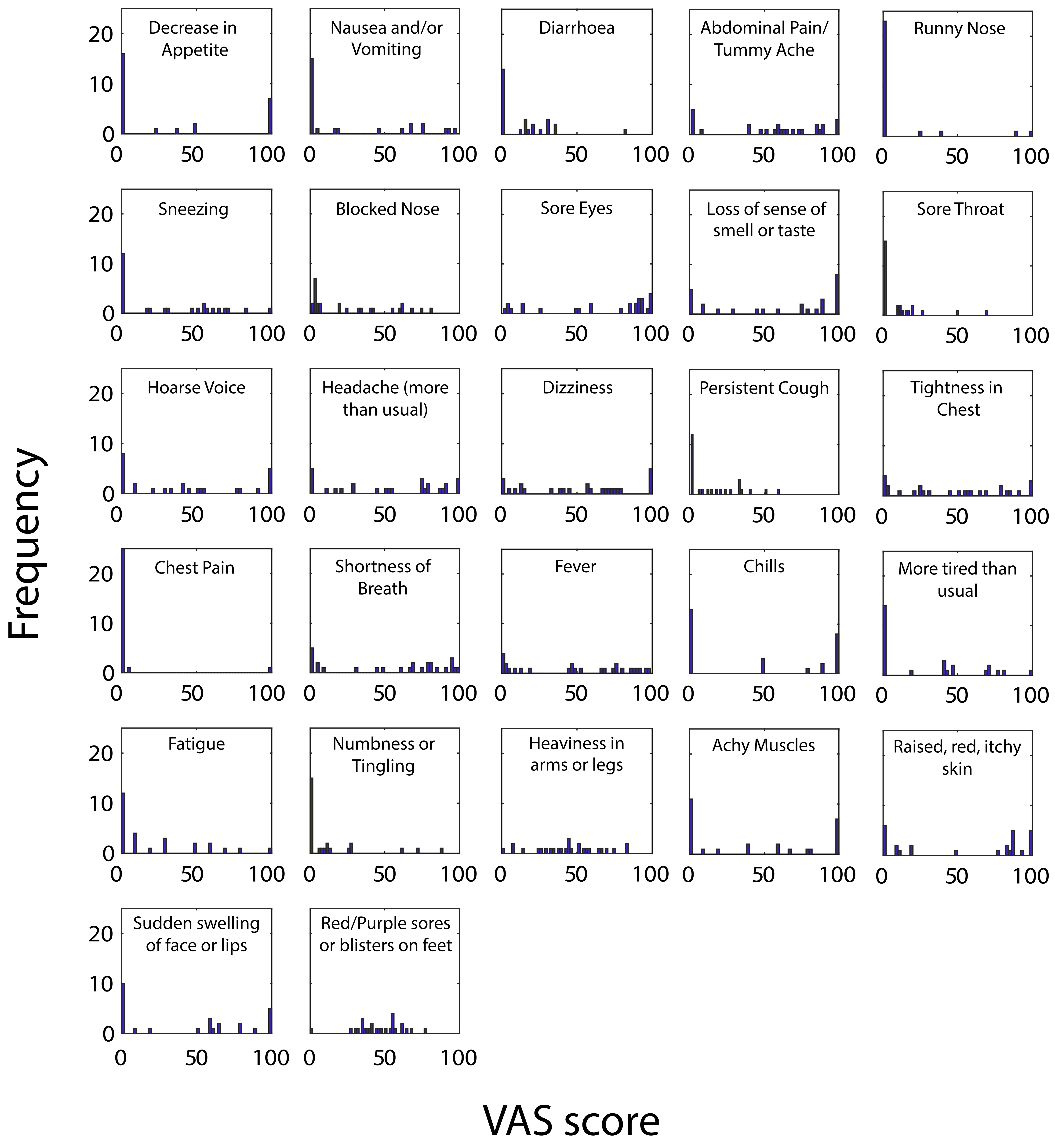

Supplement: fcae270_Supplementary_Data [file fcae270_supplementary_data.pdf]
